# Constrained Mortality Extrapolation to Old Age: An Empirical Assessment

**DOI:** 10.1007/s10680-017-9434-4

**Published:** 2017-07-24

**Authors:** Dalkhat M. Ediev

**Affiliations:** 1 Wittgenstein Centre for Demography and Global Human Capital (IIASA, VID/ÖAWWU), International Institute for Applied Systems Analysis, Schlossplatz 1, 2361 Laxenburg, Austria; 2 North-Caucasian State Humanitarian-Technological Academy, Stavropolskaya 36, Cherkessk, Russian Federation 369000; 3 Demography Department (HSMSS), Lomonosov Moscow State University, Leninskie Gory 51, r. 752, Moscow, Russian Federation 119992

**Keywords:** Old-age mortality, Life expectancy, Life table, Mortality graduation, Mortality models

## Abstract

This paper aims to improve the accuracy of parametric extrapolations of the death rates into old age by constraining the extrapolation model on presumed life expectancy at old age. Such a task is particularly important in cases where the data quality at old age, in particular the age exaggeration, is not sufficient for reliable mortality estimates. Our tests are based on period data from the Human Mortality Database and the use of the Horiuchi–Coale and Mitra formulas for reducing the bias of life expectancy in the open age interval. We show that extrapolation accuracy is substantially improved when the extrapolation is constrained by either the empirical life expectancy or the Horiuchi–Coale or Mitra estimates. Unconstrained extrapolations and those constrained by conventional life table estimates of life expectancy in the open age interval show substantial biases and should be avoided. Combining extrapolation with life expectancy estimates which are robust to the effects of age exaggeration appears to be a valuable way of improving mortality estimation.

## Introduction

Understanding mortality patterns at old age is essential for studying the processes of lifespan extension as well as population ageing and its consequences. The task is relatively straightforward for countries with well-established collection of vital statistics, although not without complications (Duthé et al. 2010; Khlat and Courbage 1996; Kibele et al. 2008; Preston et al. 1996). For populations lacking vital statistics, on the other hand, indirect estimates based on model life tables and other simplifications are commonly used to deal with data limitations. Some countries are in an intermediate situation, where vital statistics are available but suffer from inaccuracies that prevent a direct estimation of old-age mortality. Different groups and individuals have developed various approaches to overcome these data problems. The Statistics Centre of the Abu Dhabi Emirate (SCAD), for example, uses the Coale–Guo model (Coale and Guo 1989; Coale and Kisker 1990) to extend the death rates to old age and imputes the death rates at ages 85+ “based on proportions found in populations of other countries” (SCAD 2016).

Age exaggeration is a particularly difficult obstacle in establishing empirical estimates of old-age mortality. In areas where there is no tradition of documented birth registration, elderly people tend to exaggerate their age. This excludes the possibility of obtaining reliable estimates of the death rates at old ages directly from vital statistics. In Turkey, for example, where extensive data enable the calculation of detailed life tables, official estimates of old-age mortality appear to be unrealistically low (Turkish Statistical Institute 2015), possibly because of the age exaggeration. Other typical obstacles to computing death rates at advanced old age are small population sizes and the resulting erratic patterns of empirical rates at those ages (e.g. Wilmoth et al. 2007; Scherbov and Ediev 2011). In such cases, the statistical agency typically limits the analysis to death rates below the problematic age range, hence closing the official life table at some young open age interval and limiting the usability of the table. This classical method is applied in many countries where official life tables are published with rather low ages at the beginning of the open age interval (Missov et al. 2016, p. 6).

Horiuchi and Coale (1982) showed that life expectancy estimates based on life tables that are closed at a younger open age interval may be badly biased when the proportion of elderly population is growing, and suggested an adjustment formula to bypass this problem. Although Mitra (1984) questioned the Horiuchi–Coale correction and came up with an alternative formula, a more recent analysis (Ediev 2016) shows that the two methods are consistent with each other and provide a dramatic improvement in the accuracy of life expectancy estimates as compared to the classical life table with young open age interval.

**Fig. 1:**
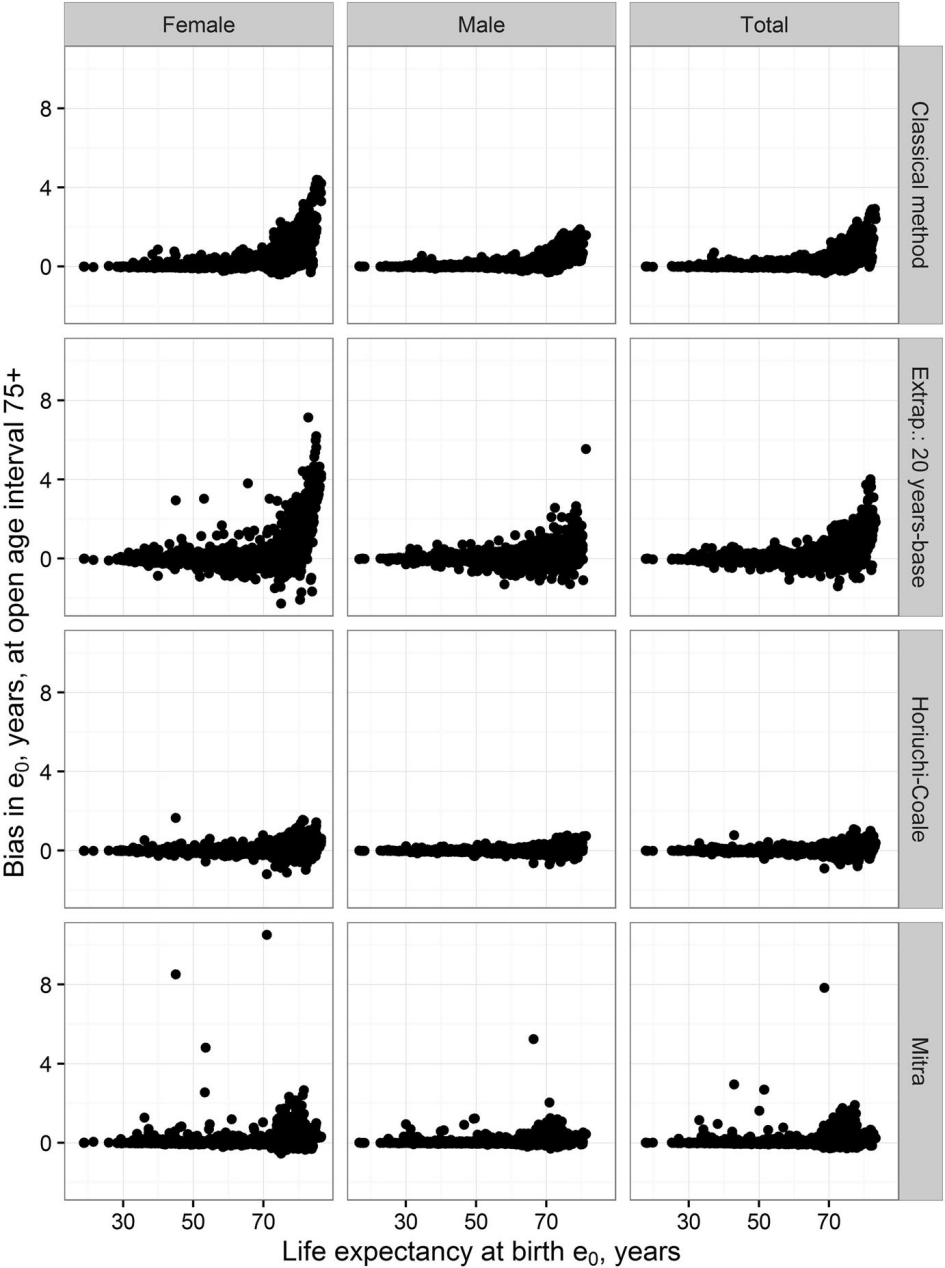
Estimation errors in life expectancy at birth, obtained by selected methods for the open age interval 75+ (in years) *Notes* Methods indicated in the right-hand side of the panels: ‘Classical method’ = traditional life table with open age interval 75+ and life expectancy for the open age interval obtained as inverse to the aggregate death rate in that interval; ‘Extrap.: 20 years-base’ = detailed life table with open age interval 110+, where the death rates for ages 75–110 are obtained by extrapolation based on their rate of change in the age range 55–74 years and the Gompertz model; ‘Horiuchi–Coale’ = same as the classical method, with the life expectancy at age 75 adjusted using the Horiuchi and Coale (1982) formula; ‘Mitra’ = same as the classical method, with the life expectancy at age 75 adjusted using the Mitra (1984) formula and our modification (Eq. 4). Source: (Ediev 2016) based on the data from the Human Mortality Database (2016)

Another common approach in dealing with problematic data at old age is to extrapolate the old-age mortality based on the death rates at younger age in combination with some mortality model, such as the Gompertz (1825) (a popular model in earlier times) or Kannisto (Thatcher et al. 1998) (the more recent favourite) models. Yet, empirical tests show that this method is not an improvement of the classical life table in terms of the accuracy of the estimated life expectancy (Ediev 2016). Selected results for the accuracy of life expectancy at birth estimated using the various methods, with the open age interval 75+, are presented in Fig. 1. The two most common approaches, the classical life table with the open age interval 75+ and the estimate based on extrapolating the death rates into the ages 75–110, produce the worst results. In fact, the extrapolation method produces even less stable results than the classical life table. Both the Horiuchi–Coale and Mitra methods, on the other hand, substantially improve the accuracy of the estimated life expectancy.

Although inferior in accuracy to the Horiuchi–Coale and Mitra methods, extrapolation is an appealing and widely used method because it produces age-specific death rates at old age. In this paper, we aim to develop a method that allows us to keep the age details of the extrapolation method while improving its overall accuracy. To this end, we use the more accurate estimates of life expectancy (Horiuchi–Coale and Mitra methods) to constrain the extrapolated rates in the open age interval. We show that such an approach leads to estimates of the death rates which are more accurate both in general in terms of life expectancy and also for individual ages.

## Data and Methods

In our study, we use the unsmoothed single-year death rates and corresponding population exposures of the Human Mortality Database (HMD) (2016) for the most recent available calendar periods for each HMD country.^1^ Altogether, the database (data downloaded on 12 February 2016) contains 46 recent country-calendar years for each gender (males, females, total). For each of the 3 × 46 = 138 input entries, we calculate life tables by assuming alternative open age intervals (the beginning age of the open age interval spanning from *a* = 65 to *a* = 85) and applying various estimation methods for life expectancy in the open age interval (described in the next paragraph). Estimates of life expectancy in the open age interval will be used to improve the extrapolations of death rates to old age.

We consider three alternative methods for estimating life expectancy in the open age interval: the classical life table model, the Horiuchi–Coale adjustment, and the Mitra adjustment. In the classical life table model (Preston et al. 2001), life expectancy is inverse to the aggregated death rate:

1 $$e_{a}^{LT} = M_{a + }^{ - 1}$$

hereafter, $a$ denotes the beginning age of the open age interval; $e_{a}$ is life expectancy at age $a$; $M_{a + }$ is the death rate in the open age interval.^2^ In the Horiuchi and Coale (1982) method, estimate (1) is adjusted for the departure of the population age composition from the stationary population assumed in the classical method:

2 $$e_{a}^{HC} = M_{a + }^{ - 1} e^{{ - \beta_{a} rM_{a + }^{{ - \alpha_{a} }} }}$$

here *r* is the annual growth rate of the population in the open age interval (to stabilize the estimates, we average the growth rate over 10-year time periods prior to the year of estimation); $\alpha_{a}$ and $\beta_{a}$ are the model parameters (for numerical values, see Horiuchi and Coale (1982) or Appendix Table 1). In the Mitra (1984) method, the adjustment involves mean population age in the open age interval:

3 $$e_{a}^{M} = M_{a + }^{ - 1} e^{{ - r\left[ {M_{a + }^{ - 1} - \left( {1 + rM_{a + }^{ - 1} } \right)\left( {\overline{x} - a} \right)} \right]}}$$

where $\overline{x}$ stands for the mean age of the population in the open age interval. Because the usage of the mean population age in (3) was questioned by Coale (1985) as prone to effects of age exaggeration, we replace it by the following regression based on HMD data (Ediev 2016):

4 $$\overline{x} = C + k_{1} M_{a + }^{ - 1} + k_{2} rM_{a + }^{ - 1}$$

where $C,k_{1} , k_{2}$ are model parameters (see Appendix Table 1 for the values).

**Table 1:** Original parameters of the Horiuchi–Coale model (2) (alpha, beta), the beta parameter of the model re-estimated on the Human Mortality Database data (Beta.hmd), and the coefficients of the mean population age model (4) estimated on HMD data (*C*, $k_{1}$, $k_{2}$)

Sex	*a*	Alpha	Beta	Beta.hmd	*C*	$k_{1}$	$k_{2}$
Female	40	1.0	0.283	0.321	50.045	0.241	−4.918
Female	55	1.1	0.207	0.241	61.025	0.303	−4.503
Female	65	1.4	0.095	0.100	69.200	0.335	−3.670
Female	75	1.4	0.095	0.109	77.701	0.380	−2.676
Female	85	1.4	0.095	0.104	86.460	0.470	−1.883
Female	95	1.4	0.095	0.062	95.591	0.626	−0.867
Male	40	1.0	0.283	0.330	50.924	0.196	−3.919
Male	55	1.1	0.207	0.236	61.406	0.269	−3.722
Male	65	1.4	0.095	0.102	69.229	0.318	−3.180
Male	75	1.4	0.095	0.108	77.563	0.379	−2.398
Male	85	1.4	0.095	0.102	86.355	0.482	−1.863
Male	95	1.4	0.095	0.058	95.633	0.609	−0.914
Total	40	1.0	0.283	0.308	50.839	0.206	−3.849
Total	55	1.1	0.207	0.234	61.115	0.293	−4.030
Total	65	1.4	0.095	0.099	69.117	0.335	−3.324
Total	75	1.4	0.095	0.108	77.583	0.387	−2.481
Total	85	1.4	0.095	0.102	86.405	0.477	−1.803
Total	95	1.4	0.095	0.061	95.518	0.658	−0.929

*Notes a* = starting age of the open age interval, *Source* (Ediev 2016)

To improve the extrapolation performance, we constrain the parameters of the extrapolation models to either the empirical $e_{a}$ or one of the estimates (1)–(3). We consider two popular mortality models that represent typical assumptions about mortality change at old age: the Gompertz and Kannisto models. Both models contain only two parameters,^3^ one of which may be determined by fixing the model death rate at the age below the open age interval, $M_{a - 1}$, to its empirical value. In the Gompertz (1825) model,

5 $$M_{x} = Ce^{{b\left( {x - a + 1} \right)}}$$

$M_{x}$ being the central death rate at age *x*, we set $C = M_{a - 1}$. In the Kannisto model (Doray 2008; Thatcher et al. 1998),

6 $$M_{x} = \frac{{Ce^{{b\left( {x - a + 1} \right)}} }}{{1 + Ce^{{b\left( {x - a + 1} \right)}} }}$$

we set $C = \frac{{M_{a - 1} }}{{1 - M_{a - 1} }}$. The second parameter, $b$, can be fitted in either model to the life expectancy in the open age interval $e_{a}$ [either the actual one or one of the estimates (1)–(3)]. We use the standard one-dimensional optimizer of the *R* package (R Core Team 2016) in finding the parameter *b* best fit to the assumed $e_{a} .$

## Results

**Fig. 2:**
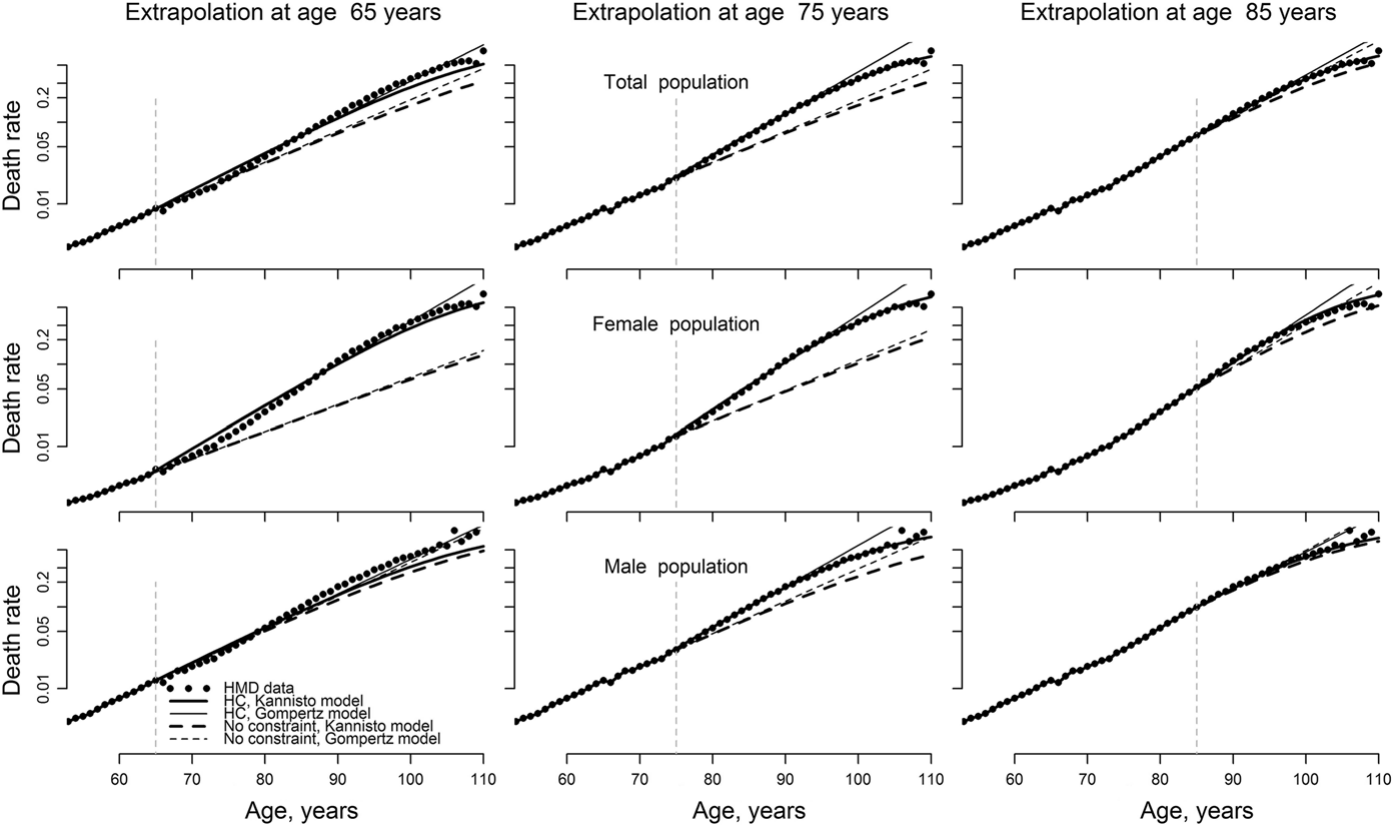
Constrained (*solid lines*) and unconstrained (*broken lines*) extrapolations of the death rates for Japan, 2012, men and women, at various ages at the start of the extrapolation (*a* = 65, 75, or 85 years as shown in the upper parts of the panels) *Notes* ‘HC’ = extrapolation constrained by life expectancy ${\text{e}}_{\text{a}}$ from the Horiuchi–Coale method; ‘No constraint’ = no constraints imposed (model parameters are estimated on the death rates for a 20-year age frame below age a); ‘HMD data’ = original death rates (points) from the Human Mortality Database (HMD 2016); ‘Kannisto model’ = death rates extrapolated using the Kannisto model at ages a+ (*thicker lines*); ‘Gompertz model’ = death rates extrapolated using the Gompertz model at ages a+ (*thinner lines*)

The potential to improve the extrapolation model by constraining its parameters is demonstrated in Fig. 2. It features extrapolations [conventional and constrained by $e_{a}^{HC}$ (2)] produced by applying the Gompertz and Kannisto models to the death rates in Japan in 2012, at three selected open age intervals (*a* = 65, 75, or 85 years). In all cases, the constrained extrapolations fit the empirical rates better than the unconstrained ones, although the improvement was small in the case of males in an open age interval 65+. In most cases, the conventional extrapolations are misleading because they produce death rates several times lower than the actual rates at old age, while the constrained extrapolations (more so the Kannisto model) stay close to the empirical curve.

**Fig. 3:**
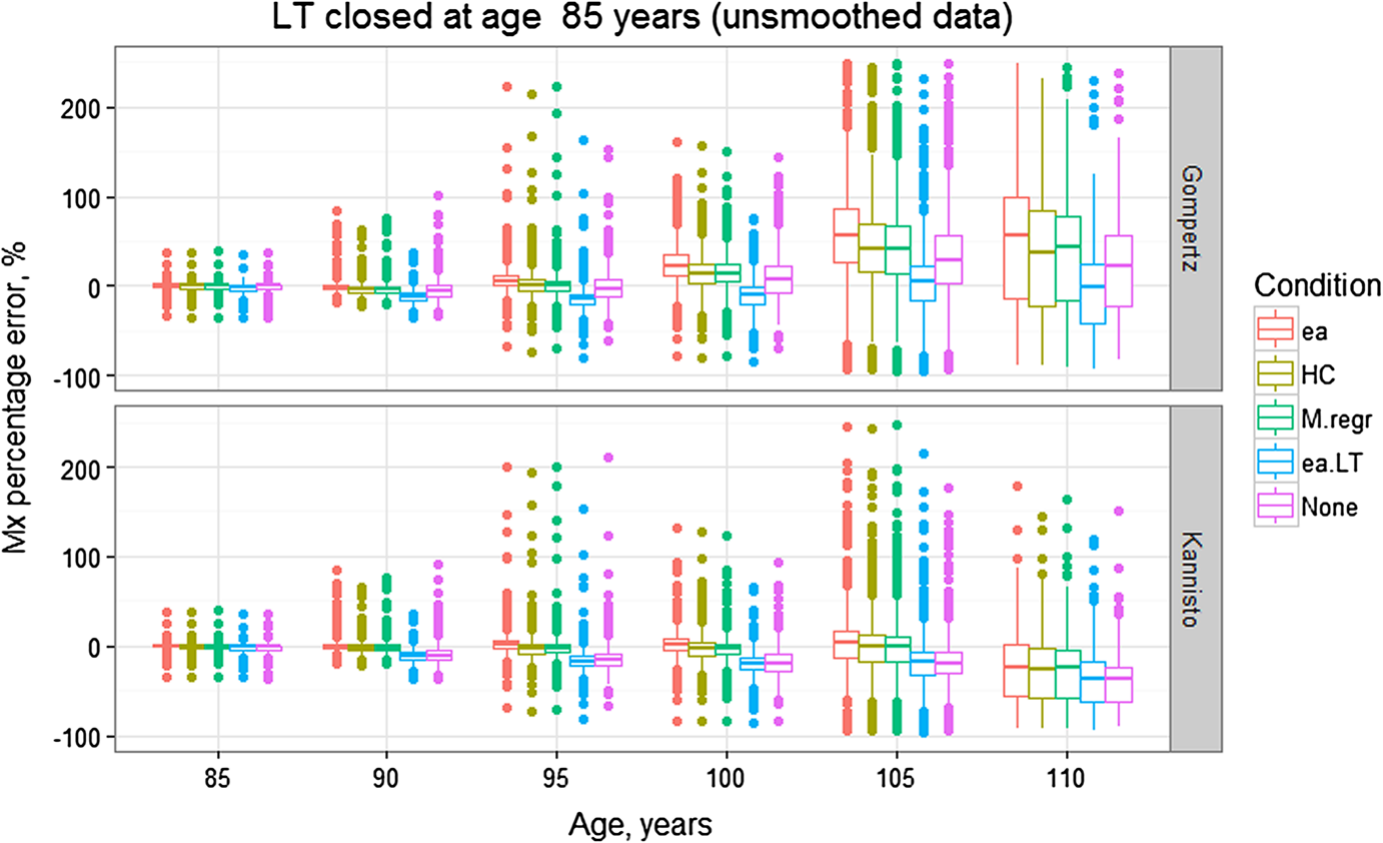
Boxplots of errors in the age-specific death rates pooled over five-year age intervals under alternative constraints imposed over the extrapolation of the death rates into the open age interval 85+ (*a* = 85) *Notes:* ‘ea’ = the extrapolation is constrained by actual ${\text{e}}_{a}$ from the HMD; ‘HC’ = extrapolation constrained by ${\text{e}}_{a}$ from the Horiuchi–Coale method; ‘M.regr’ = extrapolation constrained by ${\text{e}}_{a}$ from the modified Mitra method; ‘ea.LT’ = extrapolation constrained by ${\text{e}}_{a}$ from the conventional life table with open age interval a+; ‘None’ = no constraints imposed (model parameters are estimated on the death rates for a 20-year age frame below age *a*). ‘Gompertz’ = death rates extrapolated using the Gompertz model; ‘Kannisto’ = death rates extrapolated using the Kannisto model. Data: only the most recent available year for each country in the HMD, female, male, and total populations pooled

The example presented above is characteristic of improvements to the extrapolation method that can be achieved by constraining it to life expectancy estimates. In Fig. 3, we present the results for extrapolation errors when extrapolation starts at age 85. It features boxplots of errors surrounding age-specific death rates pooled over five-year age intervals for the Gompertz and Kannisto models. We pool the results for males, females, and both sexes combined for all HMD countries, because there appeared to be similar error patterns across different population subgroups. Extrapolations constrained by either the empirical life expectancy from the HMD^4^ or the Horiuchi–Coale and Mitra estimates are substantially more accurate, less biased, and/or more stable at ages below 97.5 for the Gompertz model and ages below 107.5 for the Kannisto model. The extrapolation constrained by the empirical life expectancy outperforms other methods at youngest age groups, as expected, although its advantage over the Horiuchi–Coale or Mitra methods fades away by about age 95. Unconstrained extrapolation and extrapolation constrained by the classical estimate (1) perform worse except at the oldest age, where the volatility of the original data seems to overshadow differences between the methods. The Kannisto model appears to better fit the age pattern of period mortality at advanced age, in terms of both the bias and the spread of errors. Counterintuitively, the constrained extrapolations [except for the one constrained by the classical estimate (1)] outperform the unconstrained extrapolation even at the youngest age interval, although the constraints should have loosened the fit of the models to data around age 85. Even constraining the extrapolation using the classical (biased) estimate of the life expectancy at the open age interval (ea.LT) somewhat stabilizes the extrapolation results, except at the very old and youngest ages.

**Fig. 4:**
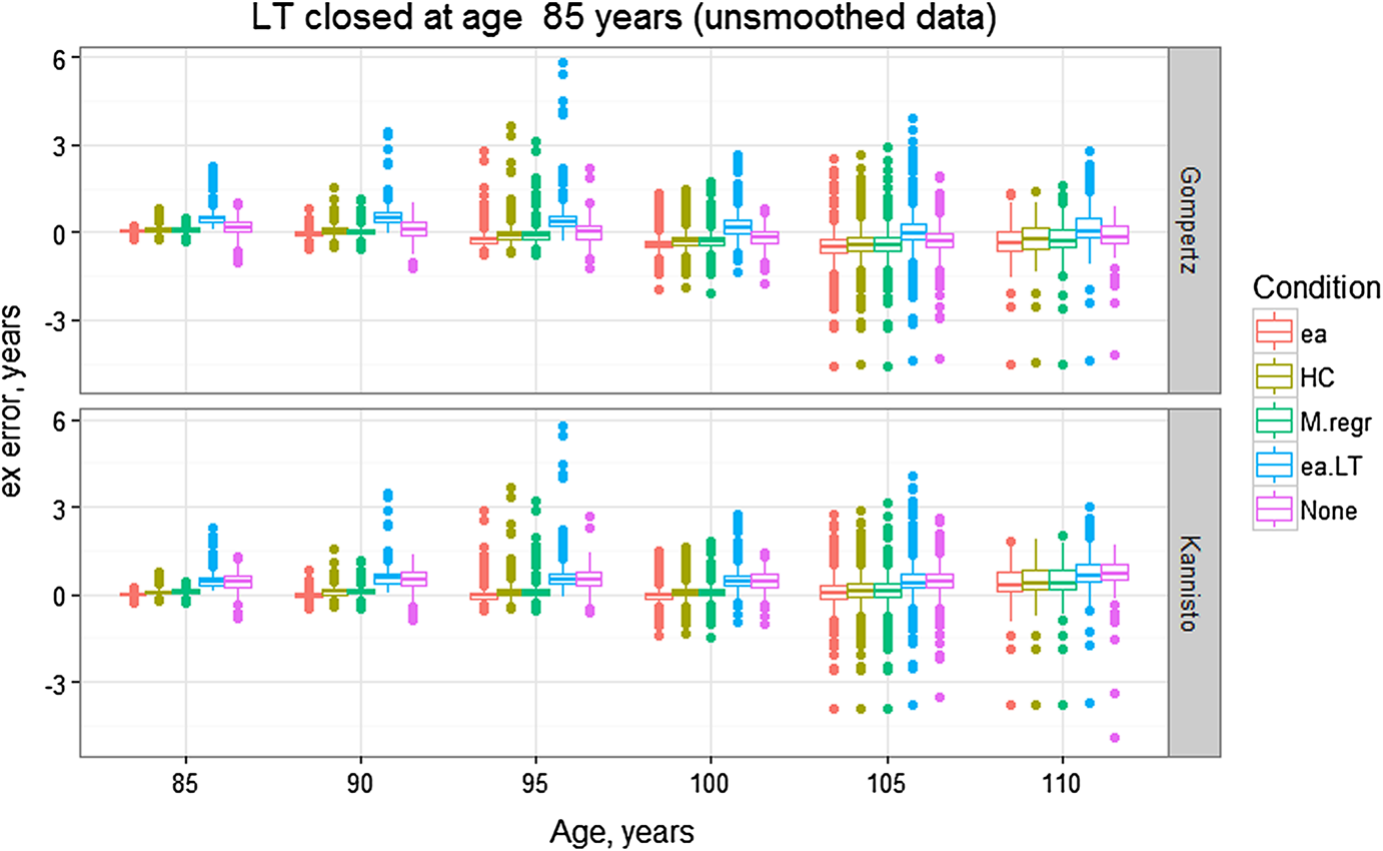
*Boxplots* of errors in remaining life expectancy by age pooled over five-year age intervals under alternative constraints imposed over the extrapolation of the death rates into the open age interval 85+ (*a* = 85) *Notes* same as for Fig. 3

Similar results apply to the errors in terms of the remaining life expectancy (Fig. 4), although the bias and instability of the conventional extrapolations are strong even at young age.

**Fig. 5:**
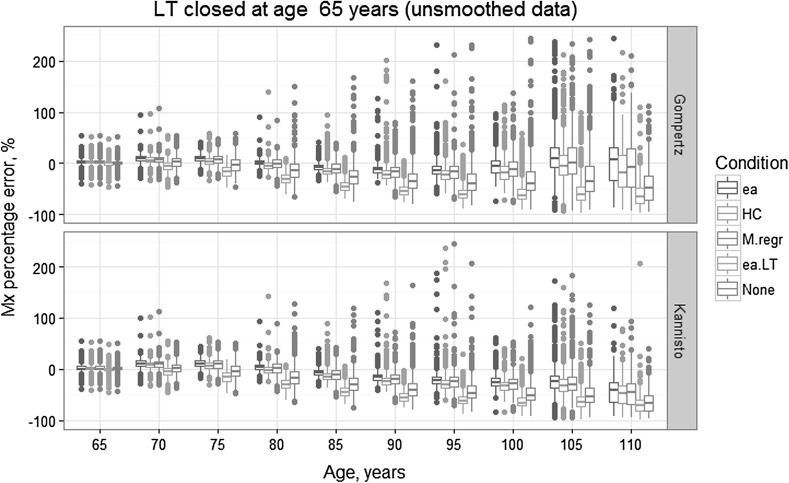
*Boxplots* of errors in age-specific death rates pooled over five-year age intervals under alternative constraints imposed over the extrapolation of the death rates into the open age interval 65+ (*a* = 65) *Notes* same as for Fig. 3

**Fig. 6:**
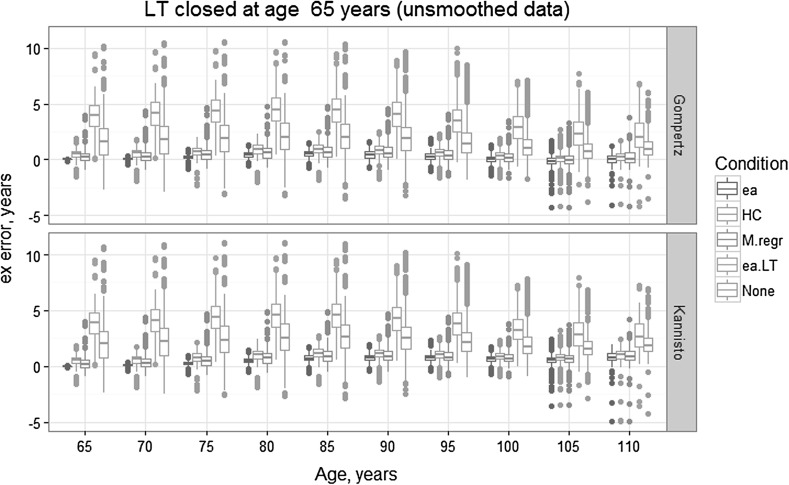
*Boxplots* of errors in remaining life expectancy by age pooled over five-year age intervals under alternative constraints imposed over the extrapolation of the death rates into the open age interval 65+ (*a* = 65) *Notes* same as for Fig. 3

Extrapolation from age 85 onwards may be a feasible option for reconstructing or graduating the death rates for countries with decent data quality below age 85 (such extrapolations used to be part of the World Health Organization’s methodology, and smoothing rates at that age are part of HMD methods protocol). It is too optimistic an option, however, for countries with poorer data, particularly with strong age exaggeration. Results which are more relevant for countries with problematic data issues are presented in Figs. 5 and 6. Here, we feature estimation errors for extrapolations into open age interval 65+. All in all, the results for the younger open age interval are similar to those presented above for the age interval 85+. However, the price for not, or wrongly, constraining the extrapolation is considerably higher. It is interesting to note that constrained extrapolations starting from age 65 and unconstrained extrapolation starting at age 85 have similar errors by age 100. Also notably, applying the ‘ideal’ constraint to actual life expectancy at age 65 provides better results than the Horiuchi–Coale and Mitra methods, throughout the entire age range up to age 105. This highlights the importance of further developing the Horiuchi–Coale and Mitra methods in order to reduce their remaining estimation biases. It can also be seen that, unlike in the case of the more advanced open age interval, the Kannisto model shows stronger systematic biases at old age when starting the extrapolation at age 65. At age 95 years and older, the bias of the (65+) Kannisto model is even stronger than that of the Gompertz model, although the wider spread of errors of the latter indicates its poorer performance. This may be taken as indication of the need to improve the mortality extrapolation models by allowing for higher flexibility of the produced mortality curve. In particular, either the three-parameter Kannisto model (Thatcher et al. 1998) or the Perks (1932) model might have offered the necessary flexibility to the mortality curve. However, our experiments with the three-parameter Kannisto and Gompertz–Makeham models including the constant background mortality term (results not shown here) did not lead to smaller biases in either model. It is also worth noting that the Kannisto model shows only small biases until age 105 when tested on HMD data for the calendar year 1970 (results not shown).

Our usage of unsmoothed raw death rates, not the smoothed life table rates from the HMD, was driven by the need to avoid possible distortions of the results by the Kannisto mortality model that was assumed when smoothing the HMD period life tables (Wilmoth et al. 2007). That same choice, however, may have increased the lack of fit of the extrapolations, especially at advanced ages where the natural stochasticity of the death rates may have dominated the differences between the extrapolations. Insight into extrapolations of death rates free of stochasticity is gained when the raw death rates are replaced by the smoothed period life table death rates of the HMD (Appendix Figs. 8 and 9). The advantage of the constrained extrapolations is even stronger and remains throughout the entire age span on the smoothed data. The Kannisto model clearly outperforms the Gompertz model on the smoothed data, although this result may be a consequence of the usage of the Kannisto model itself in smoothing the HMD rates.

## A Case Study: Old-age Mortality in Turkey, 2013/14

**Fig. 7:**
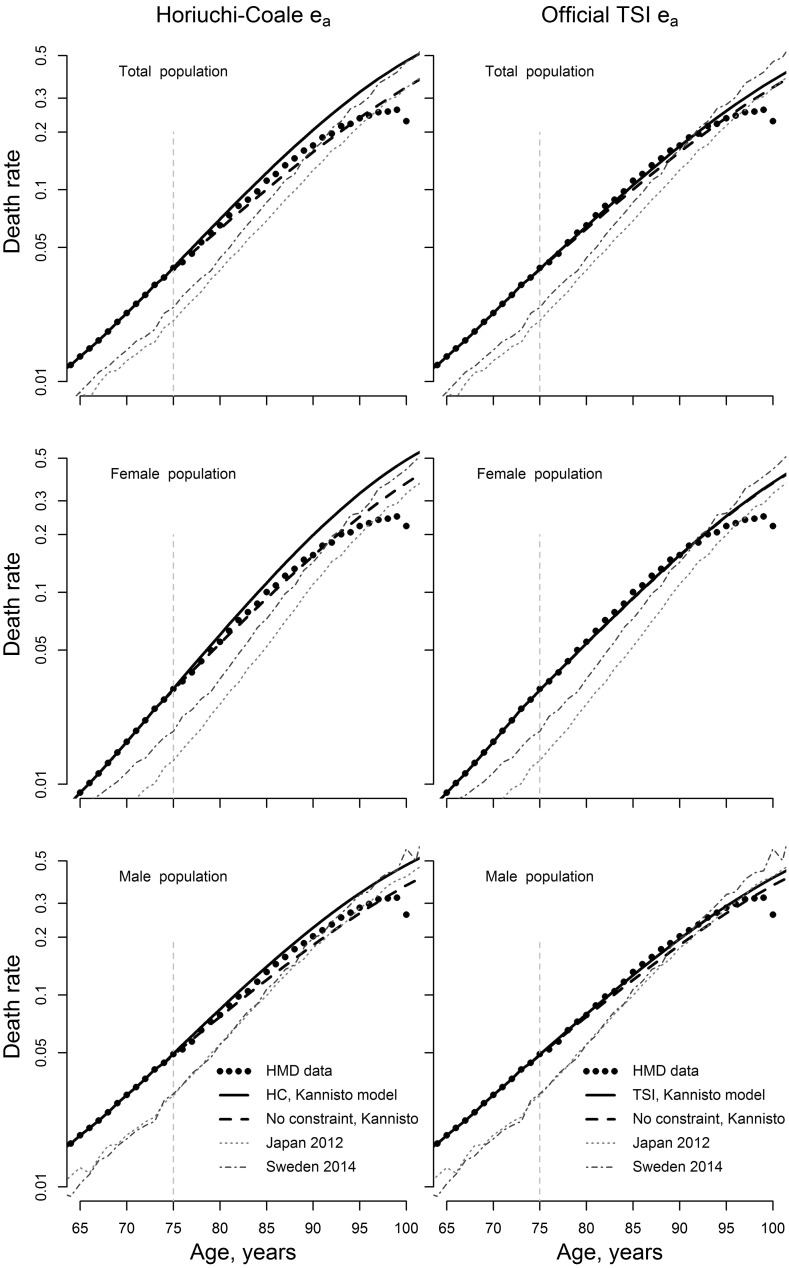
Official death rates (points) and extrapolations (*thicker black lines*) starting at age 75 years as compared to the Japanese (2012, *dotted red line*) and Swedish (2012, *dot dashed blue line*) death rates, both sexes combined. Extrapolations are based on the Kannisto model either unconstrained (*thicker broken black lines*) or constrained (*thicker solid lines*) to the Horiuchi–Coale estimate (the panels to the *left*) or to the official Turkish Statistical Institute (TSI 2015) estimates (the panels to the *right*) of the remaining life expectancy at age 75 Data: own estimates based on mortality and population data by the TSI (2015) and population growth data from the World Population Prospects (UN DESA Population Division 2015)

In this section, we supplement the general results with a case study that illustrates how substantial the necessary adjustment might be to the death rates at old age when the data are affected by age exaggeration. In Fig. 7, we present extrapolation results, from age 75 onwards, for the death rates in Turkey in 2013/14, total (the upper panels), male (the middle panels), and female (the lower panels) populations. In the case study, we constrain the extrapolations to ages 75+ to both the Horiuchi–Coale estimates of $e_{75}$ (the panels to the left in the figure) and the official estimates of $e_{75}$ by the Turkish Statistical Institute (TSI, the panels to the right; TSI assumes open age interval 100+ when constructing official life tables).

Official death rates (Turkish Statistical Institute 2015) (points in the figure) level off at unrealistically low levels at old age (compared to the recent Japanese and Swedish death rates shown in the same figure). It is quite likely that the unrealistically low official mortality rates at old age are caused by substantial age exaggeration among the elderly in Turkey.

When aggregating the death data in the open age interval 75+ and applying the Horiuchi–Coale method (population growth data come from the World Population Prospects (UN DESA Population Division 2015); mortality and population age composition data are kindly provided by the TSI), we get a remaining life expectancy e_75_ equal to 10.3, 9.4, and 11.0 years for total, male, and female populations. These are all below the official estimates of 11.0, 9.9, and 11.9 years, respectively.

Kannisto model death rates constrained to both the Horiuchi–Coale and even the official (probably subject to age exaggeration) estimates of $e_{75}$ are substantially higher at old age as compared to the official death rates. Comparing these results with rates in Japan and Sweden, the two long-time world leaders in life expectancy, it becomes clear that the official estimates of death rates at old age in Turkey must have been strongly underestimated, while the extrapolated rates look more plausible. Even the extrapolated death rates constrained to $e_{75}$ may be too low at advanced old age, as compared to the rates in Japan (more so in the case when the official $e_{75}$s are used as constraints). The unconstrained conventional extrapolations (broken lines in the figure) appear unrealistically low both at advanced old age (below Japanese and/or Swedish rates) and at younger ages where they fall below even the official estimates.

## Conclusion

The presented results confirm that conventional parametric extrapolations of death rates into old age have strong biases in terms of death rates and remaining life expectancies. These biases may be efficiently reduced when constraining the extrapolations by life expectancy in the open age interval. For instance, using the life expectancy estimated from the Horiuchi–Coale or Mitra methods provides substantial improvements in the extrapolations. Combining improved estimates of expectation of life at old age with detailed extrapolations of the age-specific death rates provides a practical tool that may be recommended in all cases where direct usage of mortality data is limited by data quality issues at advanced age. Notably, the best constrained extrapolations starting at age 65 gave errors at advanced old age that were not substantially larger than the conventional unconstrained extrapolations starting at age 85. This opens up new possibilities for correcting data that are corrupted by age exaggeration and for smoothly extending life tables to advanced old age when empirical rates show erratic patterns.

We find considerably better fit of extrapolations constrained by the empirical life expectancy at old age as compared to the extrapolations constrained by Horiuchi–Coale or Mitra estimates. This demonstrates the importance of further developing methods of estimating life expectancy at old age. One strategy to achieve this may be a recursive combination of adjustments to life expectancy and of extrapolations. While the Horiuchi–Coale and Mitra methods rely on assuming a stable population age composition, one may construct a better model of age composition by using the extrapolated death rates in the open age interval to predict unknown population exposures. Such a model may improve the accuracy of life expectancy estimates for the open age interval, and these estimates may in turn be used to improve the extrapolation model itself.

Another practical way of improving the performance of life expectancy estimates and extrapolations may be to carry out an analysis on a country basis, because age patterns of death rates and population age compositions typically bear substantial country-specific characteristics.

Our results indicate that at old age the logistic model is more stable than the Gompertz curve. Yet, the substantial systematic biases of the Kannisto model when extrapolating death rates at ages 65+ suggest that a more flexible logistic curve may provide better results for contemporary period mortality.

As mentioned in the introduction, mortality estimates for countries that lack vital statistics are usually based on indirect models, such as model life tables. These models, however, are themselves based on imputing the death rates at old age. Therefore, the model tables and old-age mortality models for developing countries may need to be revised by improving the accuracy of the underlying empirical inputs that are used in constructing those models.

Extrapolations may be useful for cohort mortality studies, but we did not explore that here. We could not examine cohort data, because the Horiuchi–Coale and Mitra methods are not suitable for that analysis. However, our results suggest that constrained extrapolation might provide a substantial improvement in accuracy for cohort mortality too. Even though the Horiuchi–Coale and Mitra models are not applicable to cohorts, using our method for cohort mortality estimates may be facilitated by the fact that the classical estimate of life expectancy (1) is accurate when cohort age structure at old age is not affected by migration and closely follows the stationary population model (Ediev 2016; Horiuchi and Coale 1982; Mitra 1984). Another promising area deserving further work is the study of extrapolation/graduation errors in constrained vs unconstrained nonparametric methods not considered here (for example, using the P-splines approach as in (Camarda 2012; Currie et al. 2004)).

With life spans expanding, policymakers and societies at large are more and more interested in understanding population change at advanced old age. Our method provides the possibility for reconstructing the numbers of people at old ages for many populations, current and historical, that lack necessary details in the original data. Accurate extrapolations may help filling gaps in studying population ageing. Reliable estimates of old-age mortality are essential for projecting the oldest old population and related needs for social welfare provisions, including healthcare that may increase dramatically at advanced ages. As shown by our case study, old-age mortality rates may be re-estimated plausibly, even without revising the official estimates of life expectancy at birth. This enables statistical agencies to adopt our method and may help increase the list of countries with reliable estimates of old-age mortality for comparative studies.

## Figures and Tables

**Fig. 8:**
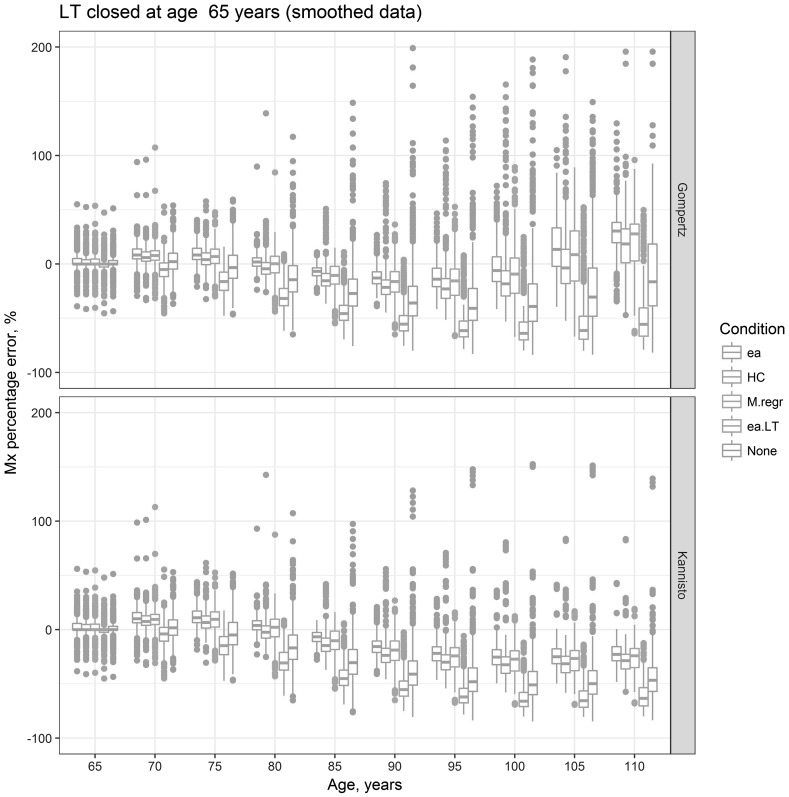
*Boxplots* of errors in age-specific death rates pooled over five-year age intervals under alternative constraints imposed over the extrapolation of the death rates into the open age interval 65+ (*a* = 65). Calculations based on smoothed HMD data *Notes* same as for Fig. 3

**Fig. 9:**
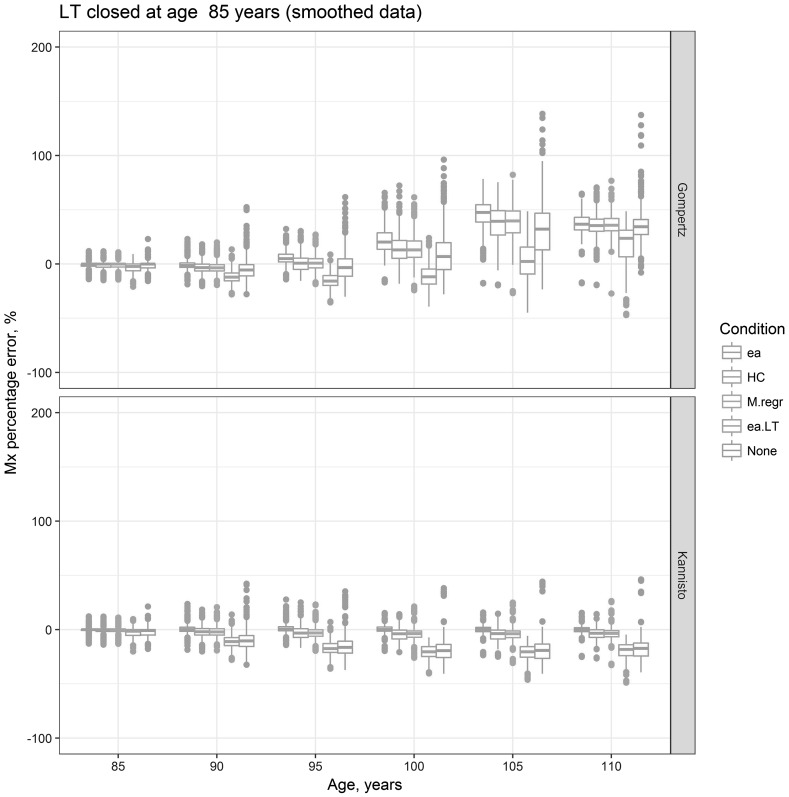
Boxplots of errors in age-specific death rates pooled over five-year age intervals under alternative constraints imposed over the extrapolation of the death rates into the open age interval 85+ (*a* = 85) Calculations based on smoothed HMD data. *Notes* same as for Fig. 3

## Appendix

Table 1 and Figs. 8 and 9.

## Footnotes

1 We present results for the most recent year in HMD for each country, although similar patterns have been obtained for earlier calendar periods too (not reported here).

2 The aggregated death rate for the open age interval is derived from the HMD (unsmoothed) death rates and population exposures: $M_{a + } = \frac{{\mathop \sum \nolimits_{x = a}^{\omega } M_{x} P_{x} }}{{\mathop \sum \nolimits_{x = a}^{\omega } P_{x} }}$, where $M_{x}$ is the death rate and $P_{x}$ is the population exposure for age $x.$

3 The two models are also known in their three-parameter variants (the Makeham (1860) model and the full Kannisto model (Thatcher et al. 1998) with the background mortality term). Those are not presented here, because inclusion of the background mortality did not change our results substantially.

4 By 'empirical' we denote the life expectancies calculated from the HMD raw data on the death rates. Because the HMD life tables are based on smoothing the raw death rates, our ‘empirical’ life expectancies may somewhat differ from the values in the HMD.
